# A Hybrid Signal Processing of RR Intervals from QTc Variation Searching Arrhythmia and Improving Heart Rate Variability Assessment in Acute Large Artery Ischemic Stroke

**DOI:** 10.1155/2016/9823026

**Published:** 2016-11-14

**Authors:** S. Rangsungnoen, P. Chanbenjapipu, N. Mathuradavong, K. Suwanprasert

**Affiliations:** ^1^Medical Engineering Program, Faculty of Engineering, Thammasat University, Bangkok, Thailand; ^2^Department of Preclinical Sciences, Faculty of Medicine, Thammasat University, Bangkok, Thailand

## Abstract

Sudden death caused by abnormal QTc and atrial fibrillation (AF) has been reported in stroke. Heart rate variability (HRV) is reduced with missing beats of RRI during arrhythmic episode and abnormal QTc variation during acute stroke. In this study, we develop a hybrid signal processing by Pan Tompkins QRS detection and Kalman filter estimator for meaningful missing beats and searching AF with prolonged QTc. We use this hybrid model to investigate RRIs of Lead II ECG in thirty acute stroke patients with long QTc and AF (LQTc-AF) and normal QTc without AF (NQTc-nonAF) and then assess them by HRV. In LQTc-AF Kalman, higher mean heart rate with lower mean RRIs compared to NQTc-nonAF Kalman was characterized. LQTc-AF Kalman showed significant increase in SDNN, HF, SD2, SD2/SD1, and sample entropy. SDNN and HF associated with high RMSSD, pNN50, and SD1 reflect predominant parasympathetic drive for sympathovagal balance in LQTc-AF Kalman. Greater SD2, SD2/SD1, and sample entropy indicate more scatter of Poincaré plot. Compared with conventional Labchart, fractal scaling exponent of *α*1 (DFA) is higher in LQTc-AF Kalman. Remarkable complexity with parasympathetic drive in LQTc-AF Kalman suggests an influence of missing beats during stroke.

## 1. Introduction

**Figure 1:**
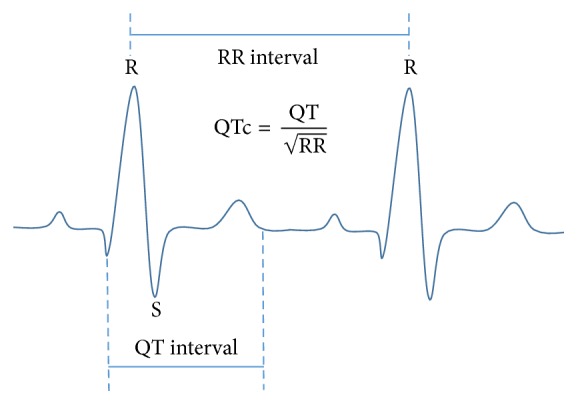
Bazett's method: divide the distance between the start of Q and the end of the T wave by the square root of the preceding RR interval.

Atrial arrhythmia (AF), prolonged QT interval, and tachycardia are the most common cardiac arrhythmia in ischemic stroke with recurrent stroke and sudden death [1, 2]. An inverse correlation between SDNN, parasympathetic HRV parameters (rMSSD and pNN50), and atrial fibrillatory rate (AFR) is reported and it is a possible link between AFR and autonomic modulation [3]. Pathophysiology of silent AF, prolonged or shortened QTc, or even ventricular tachycardia during acute stroke is poorly elucidated. Ventricular repolarization affects QT interval variation and might affect the next P wave on the second ECG cycle. R-R interval is a representative of beat to beat of heart contraction which composes of two cardiac cycles. Hence, abnormal ECG waves behave as any noise or showing missing beat are very interesting which they may influence cardiac autonomic modulation during stroke. Missing beats of R-R interval have been shown contributing abnormal QTc and arrhythmia during acute stroke. Determination of QT correction is essential for R-R interval which can be calculated in Bazett's method (Figure 1) formula that we called QTc and divided to be short, normal (NQTc), and long QT (LQTc) intervals [4]. During stroke, fluctuation of QTc is obviously found with missing beat of R-R interval. Estimation and computed missing beat of R-R interval by Kalman filter are proposed for better interpretation results with preprocessing to detect QRS complexes by Pan Tomkins algorithm as described in methodology. As we know, brain-heart axis is shifted affecting autonomic modulation in AF patient before and after stroke [3, 5–8]. These brain-heart interactions will be a new way to explore insights of sudden cardiac event and provide important information for future novel therapies to prevent recurrent arrhythmia and sudden cardiac death [2]. An effective improvement of heart rate variability (HRV) analysis is essential to verify the complexity of autonomic modulation during acute stroke [4, 9–11]. In this study, we develop a hybrid signal processing by Pan Tompkins QRS detection and Kalman filter estimator for missing beat correction in order to examine proving arrhythmia and improving HRV [12–14]. We investigate missing beat behavior in long QTc with AF and normal QTc with nonAF during 24 hours of acute stroke and then assess both groups by HRV analysis. We investigate whether QT interval fluctuation or other silent AF will be examined by hybrid signal processing method and test the difference between LQTc-AF Kalman vs NQTs-nonAF Kalman strokes. Quantitative and qualitative studies of HRV such as sample entropy, Poincare' plot, and detrended fluctuation analysis (DFA) were analyzed. We hypothesize that developed hybrid signal processing model is fitted with searching silent arrhythmia and improving HRV analysis in QTc variation.

## 2. Material and Methods

Total of 165 patients were recruited to be studied and 65 cases were characterized to be AF during acute large artery ischemic stroke by visual interpretation from physician. Thirty long QTc with AF (LQTc-AF) and no previous AF history and normal QTc without AF (NQTc-nonAF) stroke patients were investigated in this study (averaged 65 ± 4 years old and matching control). All participants gave informed consent according to local Ethic Committee (MTU-EC-IM-4-018/54). ECG data were digitized directly recording at sampling rate 1000 Hz by PowerLab system monitor. The short-term five-minute HRV was analyzed from ECG signals for linear and nonlinear HRV by using Kubios HRV software (version 2.0, University of Kuopio, Finland) [6]. Thereafter, RR intervals were examined by hybrid signal processing model before they were assessed by autonomic derangement by linear and nonlinear functions of HRV. LQTc-AF group was also examined by conventional Labchart program and compared to a hybrid model. QTc was determined and classified by Bazett's [4] and Viskin's methods [5]. QTc calculation was defined by divided distance between the start of the QRS complex and the end of the T wave with the square root of the preceding RR interval.

1. The preceding RR interval can be measured by calculating the distance between the first peak of R wave and the next peak of R wave in beats per minute.
2. The QT interval can be measured by calculating the distance between the start of the QRS complex and the end of the T wave.
3. Divide the QT interval by the square root of the RR interval.

*QT Correction (QTc) Measurement.* The QT interval was measured at a speed of 25 and 50 mm/s with the PowerLab systems (ADInstruments) and the EKGs were accepted for QT evaluation if the heart rate was between 60 and 100 beats/min. The QT interval was corrected for heart rate using Bazett's formula (QTc = QT/$\sqrt{RR}$). Short QTc (SQTc), normal QTc (NQTc), and long QTc (LQTc) were defined as lesser than 360 and 360–390 and greater than 400 milliseconds, respectively [15].

All data in linear and nonlinear functions of HRV were analyzed [9, 10]. For the processing, preprocessing by Pan Tomkins algorithm to detect QRS complexes in EKG was performed [16]. The algorithm is based on analysis of the slope, amplitude, and width of QRS complexes including a series of filters and methods that perform low pass, high pass, derivative, squaring, integration, adaptive threshold, and search procedures. The next step included applying Pan Tomkins for parameter extraction (RR interval) and then applied Kalman filter for RR interval improvement. Kalman filter algorithms have a two-step process [17]: firstly, update step measurement.

*Update.* The estimate is of value

(1) g^k−=Ag^k−1+BUk−1Pk−=APk−1AT+Q.

The Kalman filter generated estimates of the current state variables, along with their uncertainties. Secondly, the prediction step is as follows:

 
*K*_*k*_ = *P*_*k*_^−^ *H* ^*T*^(*HP*_*k*_^−^ *H* ^*T*^ + *R*)^−1^: calculation of Kalman Gain.
 
${\hat{g}}_{k}={\hat{g}}_{k}^{-}K_{k}(y_{k}-H{\hat{g}}_{k}^{-})$: estimation from measurement sensor.
 
*P*_*k*_ = (1 − *K*_*k*_ *H*)*P*_*k*_^−^: the estimate of the error covariance.

**Figure 2:**
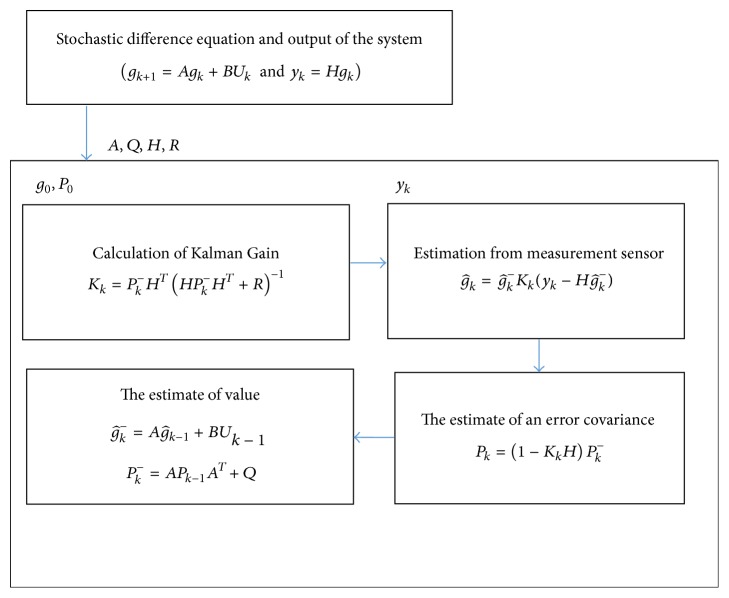
Flow chart of Kalman filter algorithm.

The outcome of the next measurement is observed; these estimates are updated using a weighted average, with more weight being given to estimates with high certainty as in flow chart (Figure 2).

## 3. Statistical Analysis

The variables are expressed as mean ± SEM. Data are analyzed by ANOVA. Significant difference is taken at *P* value <0.05.

## 4. Results and Discussion

**Figure 3:**
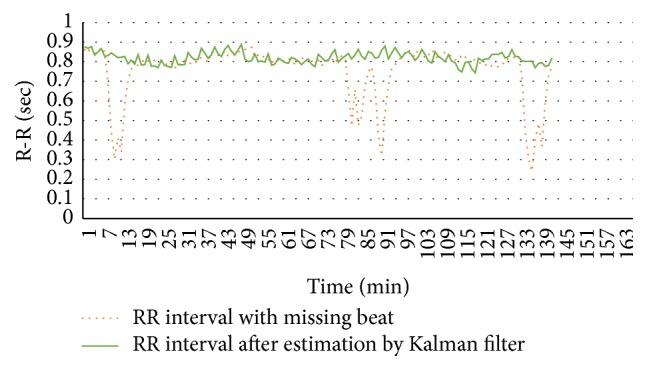
Comparison of RR with missing beat and RR after estimation.

**Figure 4:**
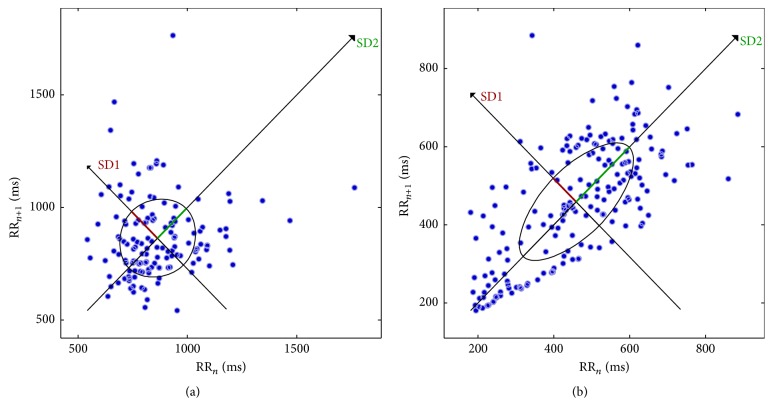
Representative case of Poincaré plot for (a) NQTc-nonAF Kalman group and (b) LQTc-AF Kalman group.

Kalman filter algorithm is demonstrated in Figure 2 which determines prediction and correction, described in methodology. Missing beats are evident in LQTc stroke case (dotted line) compared with corrected missing beats by hybrid signal processing method (solid line) as shown in Figure 3. Consistency of RRIs after Kalman estimator suggests an efficient data for further HRV analysis. In Table 1, all values show significant difference between LQTc-AF Kalman stroke and NQTc-nonAF Kalman stroke. Mean RRIs in LQTc-AF Kalman stroke are less than those in NQTc-nonAF Kalman stroke. Significantly greater parasympathetic activity is evident indicating its power drive for sympathovagal balance in LQTc-AF Kalman stroke group as shown by HF, SD2, SD2/SD1, and high value of RMSSD, pNN50, and SD1 as well [18–21]. Compared with NQTc-nonAF Kalman stroke, decreasing quantitation of sympathovagal balance is shown by lower LF/HF ratio in LQTc-AF Kalman stroke. An irregularity of signals (SamEn) in LQTc-AF Kalman stroke group is greater than in NQTc-nonAF Kalman stroke as presented by geometry of Poincaré plot in Figure 4 [19]. As in Table 2, significant difference of HRV descriptors between LQTc-AF Kalman and LQTc-AF recorded from conventional Labchart is evident. Significant difference in RMSSD, HF, SD1, SD2/SD1, and fractal scaling exponent of *α*1 indicates the impact of missing beat and the finding suggests remarkable complexity corresponding with predominant parasympathetic drive (by RMSSD, HF, SD1, and SD2/SD1) [21].

**Figure 5:**
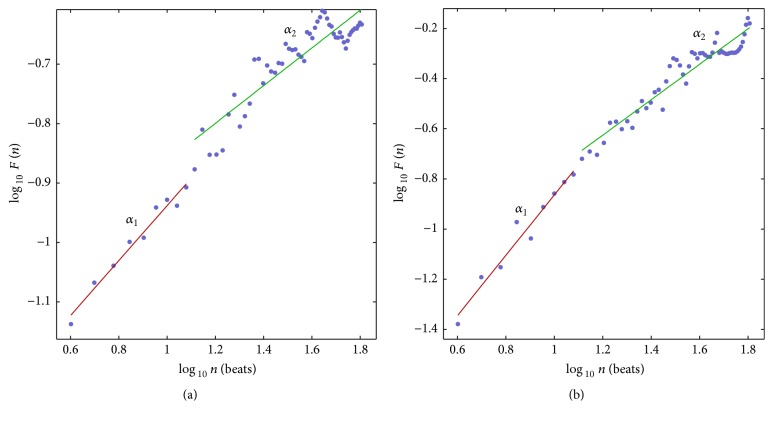
Detrended fluctuation analysis (DFA) for (a) LQTc-AF group that is not improving RR interval and (b) LQTc-AF group that is improving RR interval by Kalman filter.

DFA is more fluctuated in LQTc-AF group without improved RR interval (Figure 5(a)) compared with those in LQTc-AF group with improving RR interval by Kalman filter (Figure 5(b)). More complexity as shown by DFA in LQTc-AF Labchart group (Figure 5(a)) may be from other noise or missing beats in ECG tracing and is likely lead to poor result.

AF is a complex arrhythmia related with pulmonary vein (PV) myocardial sleeves which originally generate either reentrant or focal electrical signals. They receive parasympathetic innervation through small branches of vagus nerve [22]. It is well recognized that autonomic control is an important modulator in mediating AF [23]. Vagal stimulation causes several effects such as reducing conduction velocity in atrial tissue, shortening action potential duration and atrial effective refractory periods (ERPs), and facilitating reentrant pathway [24]. Previously, rising atrial fibrillatory rate (AFR) is inversely correlated with declining pNN50 that represents vagal outflow in acute AF stroke patient [3]. Oliveira et al. have reported that vagal activity prolongs interatrial conduction and shortens atrial and PVERP, contributing to induction and maintenance of AF in rabbit [25]. In this study, predominant parasympathetic drive in LQTc-AF Kalman may promote PV myocardial sleeves activity causing AF in arrhythmia episode in stroke. The QT interval reflects two phases of action potential in each cardiac cycle: depolarization of atrium and ventricle and repolarization of ventricle [4]. Any factors that augment depolarization or delay repolarization of the myocardium are able to increase QT interval length [5, 15]. Changes in T wave duration (ventricular repolarization) will affect the next P wave of consecutive cycle. In normal person, vagal activity reduces heart rate per minute and subsequently prolongs RRI [26]. During acute stroke, the modulation of brain-heart axis with predominant parasympathetic activity will facilitate long QTc. In summary, detection of missing beats and correction of RRI by a hybrid processing model are able to improve HRV analysis and determine AF in arrhythmia episode during acute stroke.

## 5. Conclusions

This finding suggests that a hybrid signal processing that we have developed for better RR detection by estimation of missing beats is essential in QTc variation and arrhythmia episode in stroke. Greater predominant parasympathetic activity in LQTc-AF Kalman stroke plays a vital role in sympathovagal balance of an inconsistency and irregularity signals [13, 14, 27]. High values of sample entropy corresponding with more scatter gram of Poincaré plot indicate signal turbulence and more variation in QTc with AF stroke. Compared with conventional processing, such as Labchart, our developed hybrid model fits searching silent AF and improving HRV, especially nonlinear function. Searching AF during shortened QTc episode will be further studied since it is one major cause of AF episode and acute stroke. Early detection of consistent AF with abnormal QTc variation by our hybrid model will be useful for physician to examine the stroke treatment and recurrent.

## Figures and Tables

**Table 1:** HRV comparison of long QTc-AF and normal QTc-nonAF using Kalman filter for better RR interval.

	LQTc-AF Kalman	NQTc-nonAF Kalman	^*∗*^ *P* < 0.05
Mean HR	100.30 ± 27.99	73.98 ± 12.18	*∗*
Mean RRI	669.3 ± 134.0	845.6 ± 154.5	*∗*
SDNN	114.3 ± 68.1	58.5 ± 41.3	*∗*
RMSSD	93.4 ± 48.3	34.43 ± 26.68	NS
pNN50	44.36 ± 26.55	8.10 ± 11.88	NS
VLF (0–0.04 Hz)	3001 ± 4729	1266 ± 1300	NS
LF (0.04–0.15 Hz)	6404 ± 9715	1203 ± 1733	NS
HF (0.15–0.4 Hz)	3893 ± 4022	832 ± 1234	*∗*
LF/HF ratio	1.459 ± 0.618	2.464 ± 2.501	NS
SD1	66.23 ± 34.23	24.45 ± 18.92	NS
SD2	146.9 ± 91.7	77.3 ± 54.8	*∗*
SD2/SD1	2.249 ± 0.645	3.620 ± 2.443	*∗*
SamEn	1.413 ± 0.655	0.756 ± 0.460	*∗*

NS: not significantly different. *∗*: significant difference. HR: heart rate (beat/min). RRI: RR interval (msec). SDNN (ms): standard deviation of all NN intervals. RMSSD (ms): square root of the mean squared difference between adjacent NN intervals. PNN50 (%): number of adjacent NN intervals that differ by 50 ms/total number of NN intervals. VLF (ms^2^, %): very low frequency range (<0.003 Hz). LF (ms^2^, %): low frequency range (0.04–0.15 Hz). HF (ms^2^, %): high frequency range (0.15–0.4 Hz). SD1: standard deviation of points perpendicular to the line of identity. SD2: standard deviation of points along to the line of identity. SamEn: sample entropy.

**Table 2:** HRV comparison of LQTc-AF group using Kalman filter and Labchart.

	LQTc-AF Kalman	LQTc-AF Labchart	^*∗*^ *P* < 0.05
Mean HR	100.30 ± 27.99	85.97 ± 16.74	NS
Mean RRI	669.3 ± 134.0	751 ± 139.3	NS
SDNN	114.3 ± 68.1	148.7 ± 126.3	NS
RMSSD	93.4 ± 48.3	191.9 ± 154.3	*∗*
pNN50	44.36 ± 26.55	56.64 ± 34.96	NS
VLF (0–0.04 Hz)	3001 ± 4729	8852 ± 27625	NS
LF (0.04–0.15 Hz)	6404 ± 9715	4567 ± 3878	NS
HF (0.15–0.4 Hz)	3893 ± 4022	8549 ± 7725	*∗*
LF/HF ratio	1.459 ± 0.618	0.685 ± 0.412	NS
SD1	66.23 ± 34.23	136.2 ± 109.5	*∗*
SD2	146.9 ± 91.7	159 ± 144.2	NS
SD2/SD1	2.249 ± 0.645	1.470 ± 0.704	*∗*
SamEn	1.413 ± 0.655	1.654 ± 0.474	NS
*α*1	1.0822 ± 0.1691	0.7714 ± 0.2563	*∗*
*α*2	0.8050 ± 0.234	0.6466 ± 0.2871	NS
